# The characteristics of brain function alterations in patients with chronic prostatitis/chronic pelvic pain syndrome across varying symptom severities evaluated by NIH-CPSI

**DOI:** 10.3389/fnins.2025.1511654

**Published:** 2025-02-26

**Authors:** Shengyang Ge, Yunting Xiang, Xuyun Hua, Zening Wang, Qingfeng Hu, Yijun Guo, Jingqiang Huang, Chengpeng Zhao, Jiajia Wu, Xianli Wang, Chuanyu Sun

**Affiliations:** ^1^Institute of Biomedical Sciences, Fudan University, Shanghai, China; ^2^Department of Rehabilitation Medicine, Yueyang Hospital of Integrated Traditional Chinese and Western Medicine, Shanghai University of Traditional Chinese Medicine, Shanghai, China; ^3^Department of Urology, Huashan Hospital, Fudan University, Shanghai, China; ^4^Department of Urology, Shanghai Fourth People’s Hospital, Tongji University, Shanghai, China; ^5^School of Public Health, Shanghai Jiao Tong University School of Medicine, Shanghai, China

**Keywords:** CP/CPPS, fMRI, NIH-CPSI, hierarchical clustering, chronic pain

## Abstract

**Background:**

Chronic prostatitis/chronic pelvic pain syndrome (CP/CPPS) is a prevalent condition in urology characterized by chronic pain. The pathogenesis of CP/CPPS remains unclear.

**Methods:**

We enrolled 45 eligible CP/CPPS patients and 45 healthy volunteers. We evaluated their resting-state fMRI data using a comprehensive set of parameters, such as Regional Homogeneity (ReHo) and Degree Centrality (DC), to detect brain abnormalities and identify potential correlates with the clinical manifestations of CP/CPPS. We further categorized the patients into subgroups according to their scores of NIH-CPSI to elucidate the brain changes associated with differing symptom severities.

**Results:**

Profound alterations in brain function were observed in patients with CP/CPPS. These changes involved multiple brain regions identified by DC analysis, including the right anterior cingulate cortex (ACC), left inferior frontal opercular cortex, left amygdala, right middle frontal cortex, and bilateral insula. ReHo analysis revealed significant changes in the right thalamus, left inferior frontal triangular cortex, right superior temporal pole, left ACC, and right superior frontal cortex (cluster >20 voxels, GRF correction, *p* < 0.05). Analysis using ReHo and DC revealed that brain alterations associated with varying symptom severities were localized in pain perception and modulation regions. Specifically, the DC values in the right ACC showed a linear correlation with the severity of symptoms measured by the NIH-CPSI (AUC = 0.9654, *p* < 0.0001).

**Conclusion:**

In CP/CPPS, we first discovered differences in brain function among patients with varying degrees of severity. The brain alterations of DC in the right ACC might be a potential biomarker for diagnosing and assessing disease severity.

## Introduction

Chronic prostatitis/chronic pelvic pain syndrome (CP/CPPS) is one of the most common diseases in urological outpatient practice, affecting approximately 2.2–12% of the male population throughout the world (Li et al., 2023). CP/CPPS, as its name suggests, refers to a group of chronic pain conditions characterized by invisible yet agonizing symptoms (Graziani et al., 2023). These symptoms include persistent pelvic or perineal pain, urinary disturbances such as irritative or obstructive voiding issues, sexual dysfunction, and emotional struggles like anxiety and depression (Krieger et al., 1999). Notably, these conditions could occur without any detectable urinary tract infection (Zhang et al., 2020). The clinical manifestations of CP/CPPS are characterized by persistent symptoms, refractory healing, and frequent relapses (Wu et al., 2021). In practical scenarios, CP/CPPS profoundly impacts the physical and psychological health of patients, concurrently imposing substantial financial burdens (Duloy et al., 2007).

The etiology of CP/CPPS is enigmatic. The current view holds that CP/CPPS originates from a multifaceted interplay of immunological disorders, endocrine problems, psychological factors, and dysfunctions of autonomic nervous system (He et al., 2024). Attributing to the heterogeneity of CP/CPPS, the efficacy of any monomodal therapy for CP/CPPS is predominantly inadequate (Pena et al., 2021; Qin et al., 2022). In 2009, a phenotypic classification system known as UPOINT (Urinary, Psychosocial, Organ-specific, Infection, Neurologic/Systemic, and Tenderness domains) was introduced to characterize the phenotypes of individual CP/CPPS patients and guide treatment (Bryk and Shoskes, 2021). This system provided new insight into categorizing these patients in a more nuanced approach. Moreover, the insufficient comprehension of the pathogenesis and progression of this disease also leads to the low cure rate and high recurrence rate observed following conventional therapeutic interventions (Liu et al., 2021). Certain mild central analgesics and anxiolytic/depressive medications, like duloxetine, fluoxetine, sertraline, and amitriptyline, may provide relief for associated pain by targeting the central nervous system (Zhang et al., 2020). The diagnostic protocol for CP/CPPS is essentially a process of exclusion, relying on clinical symptoms, physical examination, urine analysis, and a two- or four-glass Meares-Stamey test to exclude other potential causes like inflammatory bowel disease, acute bacterial prostatitis, prostate cancer, and so on (Pena et al., 2021; Franco et al., 2020). Considering these prevalent challenges, it is crucial to reassess our approach to understanding CP/CPPS.

Whereas the pathogenesis of CP/CPPS is multifactorial, recent evidence suggests that it may also be involved in cerebral mechanisms (Litwin et al., 1999; Dun et al., 2021; Kildegaard et al., 2019). Advanced neuroimaging techniques have catalyzed significant progress in understanding the brain-level mechanisms of CP/CPPS, transcending the focus from the prostate to the central nervous system (Coşkun et al., 2021). Given the limited cohort size, conducting in-depth analyses of the study participants was challenging, potentially restricting the comprehensiveness of the findings.

The National Institutes of Health Chronic Prostatitis Symptom Index (NIH-CPSI), as an indispensable assessment instrument, was specifically developed to facilitate self-evaluation of symptom severities and treatment responses in patients with chronic prostatitis (Litwin et al., 1999). The NIH-CPSI is structured into three principal sections with nine items. In the pain assessment segment, four items are dedicated explicitly to assessing spontaneous pain, concentrating on the location, severity, and frequency of the pain episodes. Two items are crafted to evaluate irritative and obstructive urinary symptoms in the urinary symptoms segment. In the quality of life (QoL) segment, the remaining three items are utilized to quantify the influence of these symptoms on the daily quality of life, capturing the broader implications for patients’ functional well-being. The NIH-CPSI, now available in multiple languages, is a reliable, effective, and pivotal instrument for evaluating the symptom severity and therapeutic efficacy of prognosis in managing chronic prostatitis (Dun et al., 2021; Kildegaard et al., 2019; Coşkun et al., 2021).

Herein, we continued to inspect the potential brain functional changes associated with CP/CPPS. We assessed their resting-state fMRI data by using a comprehensive set of parameters to detect subtle neurological differences and identify potential correlates with the clinical manifestations of CP/CPPS. Furthermore, to enrich our analysis, we categorized these patients of CP/CPPS into various subgroups founded on their scores of NIH-CPSI to facilitate a nuanced analysis of brain changes associated with differing symptom severities. By examining the fMRI data through the degrees of symptom severity, we aimed to uncover the central neural mechanism that may contribute to the heterogeneity observed in CP/CPPS patient outcomes.

## Materials and methods

### The recruitment of participants

In this research, we recruited 45 male right-handed patients experiencing spontaneous pelvic pain triggered by CP/CPPS and another 45 right-handed, healthy participants, carefully matched for age and gender, to form a comparative cohort of healthy controls (Table 1). Patients with CP/CPPS were initially recruited through oral inquiries at the urology outpatient clinic of Huashan Hospital, Fudan University, and were provided with comprehensive details about the research plan. Concurrently, healthy controls were recruited through online and social media advertisements. This study was conducted under the approval and supervision of the Ethics Committee of Huashan Hospital, Fudan University (Ethics Approval No. 2023–851) and the Ethics Committee of Jing’an District Central Hospital, Fudan University (Ethics Approval No. 2020–05), ensuring strict compliance with ethical research standards. To mitigate the potential impact of age-related brain atrophy and exhibit a more homogenous group for evaluating cerebral dynamics unrelated to senescent changes, all participants were recruited within the age range of 20 to 50 years (Khachatryan and Robinson, 2018). Based on the chief symptoms, thorough physical examinations, routine urinalysis, urine microbiological cultures, and transrectal ultrasonography (TRUS), the patient’s diagnosis of CP/CPPS was established by following the standardized diagnostic procedures. Following the diagnostic criteria for CP/CPPS and to exclude individuals with mild symptoms, all enrolled patients were required to report chronic pelvic pain persisting for more than 3 months. Furthermore, extra potential diseases that could affect the study’s outcomes were rigorously excluded, like acute or chronic bacterial prostatitis, prostate cancer, benign prostatic hyperplasia, and other abdominal or pelvic floor syndromes. The eligibility of participants was further refined by excluding individuals who claimed other chronic pain disorders, malignant tumors, or chronic disorders known to induce peripheral nerve damage, such as hypertension, Guillain-Barré syndrome, and diabetes mellitus. The enrolled patients of CP/CPPS either denied taking any medications or alternative treatments for CP/CPPS before this research or had ceased any therapies for treating CP/CPPS for over a month. Those participants reported no discomfort in other parts of their bodies. Contraindications for the fMRI scan, such as claustrophobia and the presence of dental implants, were also taken into account.

**Table 1:** The characteristics of participants.

	Patients of CP/CPPS	Health control	*p*-value
Number of participants	45	45	/
Gender	MALE		/
Age	37.250 ± 9.0234	35.852 ± 9.236	0.316
NIH-CPSI total scores		/	
Total Scores	28.890 ± 9.583	/	
Pain and discomfort (item 1 + 2 + 3 + 4)	15.255 ± 6.467	/	/
NRS (item 4)	4.909 ± 1.956	/	/
Lower urinary tract symptoms (item 5 + 6)	4.836 ± 3.143	/	/
Impact on quality of life (item 7 + 8 + 9)	8.945 ± 2.408	/	/
Severity of symptoms (item 1 + 2 + 3 + 4 + 5 + 6)	20.091 ± 7.791	/	/

### The acquisition of fMRI data

Before the fMRI scanning, all participants were requested to sign the written informed consent forms, following an exhaustive explanation of the research’s procedures and their implications. After signing the written informed consent forms, those participants were instructed to complete the NIH-CPSI questionnaire, facilitating an assessment of the subjective severity and impact of their symptoms. In the group of CP/CPPS patients, to guarantee the accuracy of the fMRI data in relation to their symptomatic state, only individuals experiencing chronic spontaneous pain during the entire scanning session were selected to participate. The acquisition of resting-state fMRI data was conducted utilizing a 3.0T GE MR 750 MRI scanner which was equipped with an 8-channel phased array head coil in the Jing’an District Central Hospital, Fudan University. The data was procured using a gradient-recalled echo-planar imaging pulse sequence with the following settings: TR/TE = 2,000/30 ms; FA = 90°; acquisition matrix = 64 × 64; FOV = 24 × 24 cm^2^; slice thickness = 4 mm; 38 slices with 210 total time points. The high-resolution T1-weighted MRI images were taken with a three-dimensional fast spoiled gradient-echo dual-echo sequence (specifics: TR = 8.2 ms; TE = 3.2 ms; matrix = 256 × 256; FOV = 24×24 cm^2^; slice thickness = 1 mm with 156 slices). To precisely categorize the participants, the authors had access to individual information either during or subsequent to the data acquisition process. Throughout the acquisition, preprocessing, processing, and analysis of the fMRI data, at least one senior neuroradiologist was engaged to ensure and evaluate the imaging quality, safeguarding the integrity and reliability of the neuroimaging assessments.

### The preprocessing of fMRI data

Data preprocessing was meticulously executed using the RESTplus V1.22 toolkit^1^ in conjunction with the Statistical Parametric Mapping 12 (SPM12) toolbox^2^. To begin with, DICOM images were converted into NIFTI format, followed by the exclusion of the first 10 time points to mitigate initial instability. Then, slice timing was applied to correct for temporal discrepancies between image layers. The images were spatially realigned through rigid-body transformations to adjust for misalignments caused by head motion, with data exhibiting excessive motion (surpassing 2° of maximum rotation and/or 2 mm of maximum displacement) excluded to maintain the data quality. Spatial normalization to the standard Montreal Neurological Institute (MNI) EPI template was performed, resampling the images to isotropic 3 mm x 3 mm x 3 mm voxels with normalization parameters. Afterwards, the processed images were smoothed with a 6 mm full width at half maximum (FWHM) Gaussian kernel to enhance signal uniformity. This step of spatial smoothing was specially excluded for the calculation of ReHo to avoid data being repeatedly smoothed. The linear tendency within the preprocessed data was eliminated via linear regression. The nuisance covariates, including head motion parameters, white matter signal, and cerebrospinal fluid signal, were regressed out to minimize their confounding effects. Ultimately, the voxels were subjected to temporal bandpass filtering (0.01–0.08 Hz) to attenuate the impact of low-frequency drifts and high-frequency physiological noises.

### SmKCC-ReHo analysis of fMRI data

Regional Homogeneity (ReHo) analysis was conducted using the RESTplus V1.22 software following a rigorous data preprocessing pipeline, which was performed using an isotropic Gaussian kernel with a 6-mm FWHM, as previously described (Lu et al., 2024). The Kendall’s coefficient concordance (KCC) method was employed to assess the temporal similarity of the time series between each voxel and its 26 adjacent voxels. Once the ReHo maps for each subject were computed, they were normalized by dividing by the subject’s mean whole-brain ReHo value, which reduced variability due to individual differences. Subsequently, we derived each participant’s mean KCC-ReHo (mKCC-ReHo) maps. All these maps were refined by further smoothing with a Gaussian kernel of 6-mm FWHM, culminating in smoothed mean KCC-ReHo (SmKCC-ReHo) maps.

### Degree centrality analysis of fMRI data

We also utilized the RESTplus toolbox to compute the weighted degree centrality (DC) measures, a key metric in network analysis. Initially, the time series data for each voxel were extracted. Subsequently, we calculated the Pearson’s correlation coefficients (*r*) between each voxel’s time series and those of all other voxels within a gray matter mask, yielding a comprehensive whole-brain functional connectivity (FC) matrix for each participant with a threshold set at *r* > 0.2. Individual correlation matrices were transformed into *Z*-scores using Fisher’s *r*-to-*z* transformation to enhance the normality of the data. Finally, the weighted DC strength for a voxel was determined by summing the *Z*-values of its connections with all other voxels, thereby quantifying its regional centrality and characterizing the pivotal roles within the brain’s functional network.

### Statistics analysis

Demographic data, specifically the ages of participants, were compared between patients with CP/CPPS and healthy controls using two-sample *t*-tests. The correlation of items in the NIH-CPSI was calculated. Significance was set at *p* < 0.05. Additionally, two-sample *t*-tests were conducted on the ReHo and DC values between the CP/CPPS and control groups using RESTplus software integrated with SPM 12 (Jia et al., 2019). Gaussian Random Field (GRF) theory was employed for multiple comparison correction, adjusting for ReHo and DC values at voxel *p* < 0.01 and cluster *p* < 0.05. The threshold for statistical significance was set at *p* < 0.05.

To illustrate brain functional changes at different stages of symptom progression, we categorized the enrolled CP/CPPS patients into distinct groups based on the severity of symptoms, specifically severe, moderate, and mild, as indicated by their NIH-CPSI scores. Subsequently, we performed independent comparisons to analyze brain functional alterations across these groups. Two-sample *t*-tests were utilized for pairwise comparisons to assess differences among the groups, with statistical significance defined as *p* < 0.05. The comparative results were visually presented using a colored heatmap for improved data interpretation.

We designated all abnormally activated brain regions as regions of interest (ROIs) and extracted the voxel values from these ROIs. Corresponding voxel values from the homologous ROIs were extracted. To unveil the diagnostic potential of specific brain regions within predefined ROIs according to the DC and ReHo analysis, we launched ROC analysis to determine the sensitivity and specificity of each region in distinguishing between healthy controls and patient groups. We constructed the Receiver Operating Characteristic (ROC) curves by plotting the true positive rates against the false positive rates for groups of patients and health controls. Then, we calculated the Area Under the Curve (AUC) for each curve to evaluate the significant difference with *p* < 0.05.

Additionally, the extracted voxel values of ROIs were correlated with the NIH-CPSI scores of the respective CP/CPPS patients. A simple linear regression analysis was conducted to establish these correlations. The results were graphically depicted using GraphPad Prism version 10.0, with statistical significance set at *p* < 0.05.

## Results

### The demographic and clinical features of enrolled participants illustrated significant differences between patients with CP/CPPS and health control

As shown in Table 1, the features of our recruited groups involved both patients with CP/CPPS (*n* = 45) and healthy volunteers as the control group (*n* = 45), who were male, right-handed, and age-matched (*p* = 0.316). To mitigate the influence of age-related brain atrophy on our study outcomes, we established an age criterion for participant recruitment, limiting eligibility to individuals within the 20–50 years age bracket. The baseline of average duration with the symptom of spontaneous chronic pelvic pain was about 20 months, consistent with the diagnostic criteria for CP/CPPS. The mean scores of total NIH-CPSI were slightly over 28.890 ± 9.583, reflecting a moderate severity of symptoms among the participants of this study. Since this score exceeding 18 on the NIH-CPSI characteristically indicated a significant level of discomfort and diminishing in patients’ QoL, in this cohort, we found there would be a considerable influence on these individuals with scores above 28. The scores on the Numerical Rating Scale (NRS) in the patient group were 4.909 ± 1.956, signifying a moderate to severe level of pain intensity. The NRS served as an essential instrument in our assessment process, effectively aiding in excluding potential chronic pain patients and ensuring the precise determination of eligibility for healthy volunteer participants. Furthermore, the Pain and Discomfort scores were 15.255 ± 6.467, presenting a significant but variable level of discomfort among the participants. Similarly, the item Severity of Symptoms scores were 20.091 ± 7.791, revealing a moderate to severe impact of symptoms on participants. The inherent heterogeneity of CP/CPPS likely accounts for this observed phenomenon, suggesting the necessity to further categorize and understand the diverse presentations within this complicated syndrome. Hence, in the fMRI data analysis procedure, each participant’s fMRI data was stratified into three distinct groups based on the severity of their symptoms, as determined by their total scores on the NIH-CPSI. By employing this stratification, we customized our data analysis strategies to further investigate the potential brain alterations associated with the development of chronic prostatitis-like symptoms within each subgroup of CP/CPPS patients.

### The altered brain regions between CP/CPPS patients and health control detected by DC and ReHo

**Figure 1:**
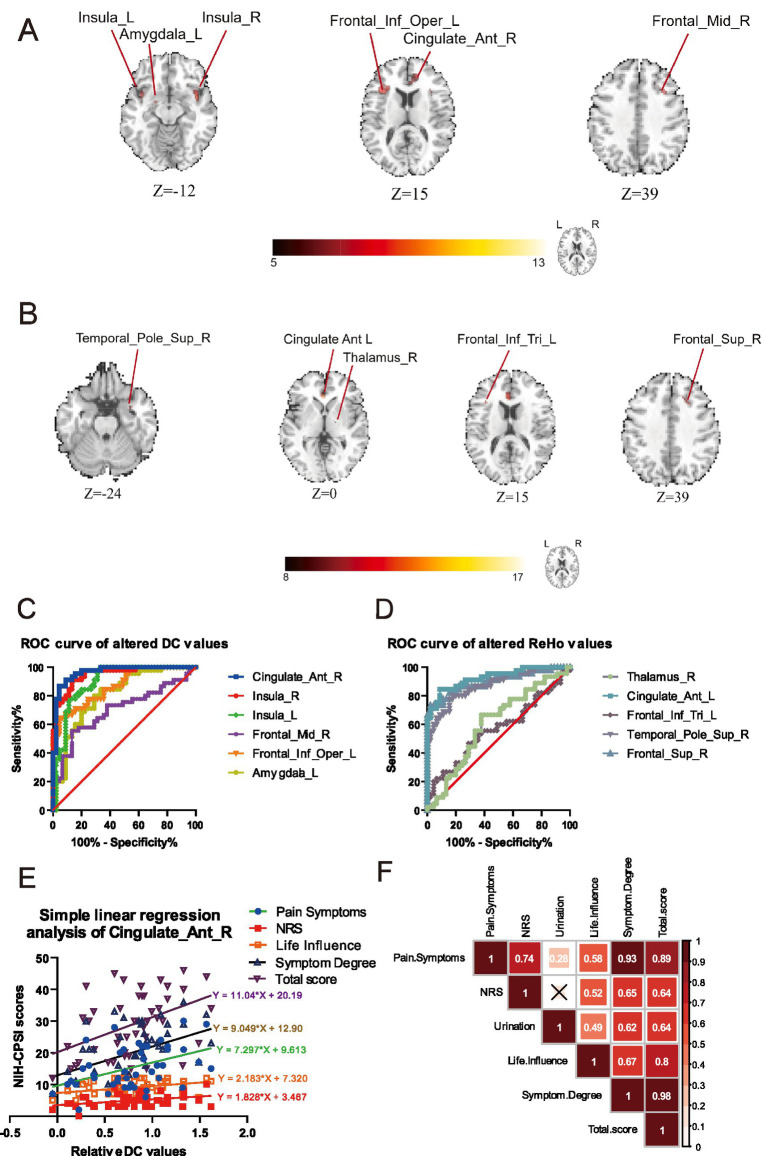
The analysis of DC and ReHo detected the functional brain alterations between the groups of CP/CPPS patients and healthy volunteers. **(A)** The alterations of DC compared by CP/CPPS patients and health control. **(B)** The alterations of KCC-ReHo compared by CP/CPPS patients and health control. **(C)** The analysis of ROC revealed the altered brain regions of right ACC and right insula had the potential predictive value of diagnosis evaluated by the DC values. **(D)** The analysis of ROC showed a poor predictive value of diagnosis assessed by the ReHo values. **(E)** Simple linear regression analysis of several NIH-CPSI item scores with DC values in the brain regions of right ACC. **(F)** The correlation analysis of NIH-CPSI item scores exhibited significant internal associations.

In Figures 1A,B, we observed the abnormal neural activities among the cohort of patients with CP/CPPS, compared with the health control. These brain functional alterations were likely associated with pain perception and emotional regulation. As listed in Table 2, abnormal positive neural activities in the patients of CP/CPPS got involved in the brain regions of right anterior cingulate cortex (ACC), left inferior frontal opercular cortex, left amygdala, right middle frontal cortex, and left and right insula (cluster>20 voxels, GRF, *p* < 0.05) by the analysis of DC. Likewise, in Table 3, there were positive brain abnormalities found in the brain regions of the right thalamus, left inferior frontal triangular cortex, right superior temporal pole, left ACC, right superior frontal cortex, and right superior frontal cortex (cluster>20 voxels, GRF, *p* < 0.05) detected by ReHo analysis. These alterations indicated aberrant neural activity within the pain modulation circuitry, implying that individuals with CP/CPPS might exhibit a dysregulated pain modulation system.

**Table 2:** The brain regions of abnormal activities assessed by the analysis of DC.

Contrast name				MNI Coordinates		
	Region label	Extent	*t*-value	*x*	*y*	*z*
Positive	Cingulate_Ant_R	500	12.594	3	30	0
	Frontal_Inf_Oper_L	241	7.097	−36	12	15
	Amygdala_L	7	6.940	−18	0	−12
	Frontal_Mid_R	47	7.965	33	18	39
	Insula_L	240	7.834	−42	6	−12
	Insula_R	494	10.792	42	3	−12

**Table 3:** The brain regions of abnormal activities assessed by the analysis of ReHo.

Contrast name				MNI Coordinates		
	Region label	Extent	*t*-value	*x*	*y*	*z*
Positive	Thalamus_R	19	9.015	15	−6	0
	Frontal_Inf_Tri_L	77	12.609	−36	21	15
	Temporal_Pole_Sup_R	51	10.622	36	3	−24
	Cingulate Ant L	768	16.657	0	27	−3
	Frontal Sup R	80	11.966	24	18	39

### The ROC analysis clarified the diagnostic significance of regional brain activity in ROIs

**Figure 2:**
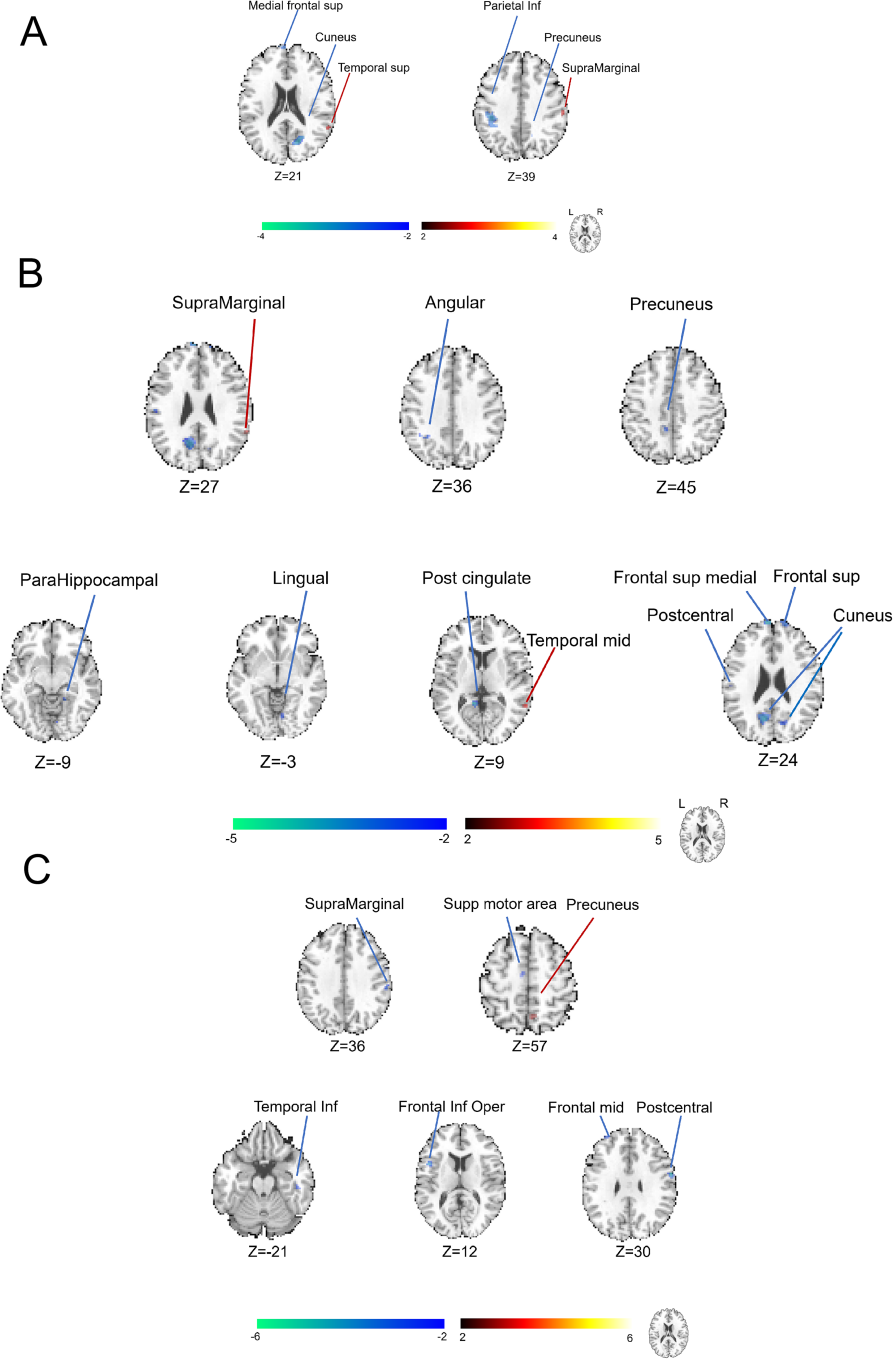
Categorization of brain functional alterations identified by DC analysis in patients with CP/CPPS stratified by symptom severity. **(A)** Comparison between the groups of severe and moderate conditions with symptoms. **(B)** Comparison between the groups of moderate and mild conditions with symptoms. **(C)** Comparison between the groups of severe and mild conditions with symptoms.

In Figure 2C, we discovered that the AUC for the DC values in the right ACC exhibited notably robust reliability, with an AUC of 0.9654 (*p* < 0.0001), indicating a substantial ability to discriminate between groups. Furthermore, the AUC of the right insula also displayed profound diagnostic potential, with an AUC reaching 0.9506 (*p* < 0.0001). In Figure 1F, we demonstrated that specific DC values within the right ACC revealed a significant degree of dispersion, indicating reliability in this neural model of distinguishment. Nevertheless, there was no other brain region with the AUC exceeding 0.95. The results of ROC by the altered ReHo values exhibited a less pronounced diagnostic capability compared to these key regions of interest by the altered DC values.

### The linear regression analysis of voxel intensities with the scores of NIH-CPSI elucidated the intrinsic tendency of CP/CPPS

Through a straightforward linear regression model, we aimed to quantify the association between the neuroanatomical changes and the self-reported symptoms measured by the NIH-CPSI. We observed a consistent trend indicating a linear increase in the DC values within the right ACC about the severity of various symptom items, except for urinary symptom scores. In the Figure 1E, we found there was statistically significant linear regression between the relative DC values and the NIH-CPSI Pain Symptoms (slope: 7.297, *R*^2^ = 0.1921, *p* = 0.0026), NRS (slope: 1.828, *R*^2^ = 0.1323, *p* = 0.0140), NIH-CPSI Life Influence (slope: 2.183, *R*^2^ = 0.1406, *p* = 0.0112), NIH-CPSI Symptom Degree (slope: 9.049, *R*^2^ = 0.1965, *p* = 0.0023) and NIH-CPSI Total Score (slope: 11.04, *R*^2^ = 0.1968, *p* = 0.0023). The deviation from the general trend suggested a complex interplay of factors influencing symptom progression, delineating the heterogeneity in how patients experienced and reported their symptoms over time.

### The correlation analysis of each item in the NIH-CPSI illuminated possible relationships between symptom dimensions

In Figure 1F, to further investigate the interrelationships among the various symptom domains, we examined the relationships among various dimensions of symptoms as measured by NIH-CPSI. There was a strong correlation (*r* = 0.98) between the Total Score and Symptom Degree, attributing to the significant proportion of the total score accounted for by symptom degree. Meanwhile, it was found that there was a high positive correlation (*r* = 0.93) between Pain Symptoms and Symptom Degree, suggesting that an increase in pain severity was closely associated with the self-reported pain symptoms. It was apparent that the lowest correlation coefficient (*r* = 0.28) was observed between Urinary Symptoms and pain Symptoms, which revealed a relative lack of association between these two distinct sets of chief complaints. Compared with the correlation (*r* = 0.49) between Life Influence and the severity of Urinary Symptoms, a moderate correlation between Life Influence and Pain Symptoms (*r* = 0.58) was also detected, indicating that the patient’s quality of life was more readily affected by pain symptoms. Given the small sample size, these correlations might exhibit some limitations.

### The DC analysis presented extra comparative brain functional changes among groups defined by the symptomatic severity of CP/CPPS

To elucidate the additional comparative brain functional alterations, we applied the DC analysis, another parameter in the analytic procedure of fMRI data. In Figure 2A and Supplementary Table S1, the brain regions of the left middle cingulate gyrus and left lingual gyrus (cluster>5 voxels, GRF, *p* < 0.05) were discovered to display some decreased abnormalities of DC values. In Figure 2B and Supplementary Table S2, the brain regions, including the left superior temporal gyrus, right lingual gyrus, left cuneus, right superior parietal lobule, left postcentral gyrus, left inferior parietal lobule and left superior parietal lobule (cluster > 5 voxels, GRF, *p* < 0.05) were found to exhibit increased abnormalities in DC value. In the meantime, the brain regions of the left olfactory cortex, left precentral gyrus, right putamen, right middle frontal region (area 2), left anterior cingulate cortex, left caudate nucleus and right postcentral gyrus (cluster > 5 voxels, GRF, *p* < 0.05) showed decreased abnormal alterations in DC values when comparing groups between moderate and mild conditions with symptoms. In Figure 2C and Supplementary Table S3, the brain regions of the left cuneus, right precuneus and right cuneus were observed to be abnormal with negative activations, indicating heightened neural activity within these areas.

### The analysis of KCC-ReHo revealed the separate brain functional abnormalities associated with varying degrees of symptom severity evaluated by NIH-CPSI scores

**Figure 3:**
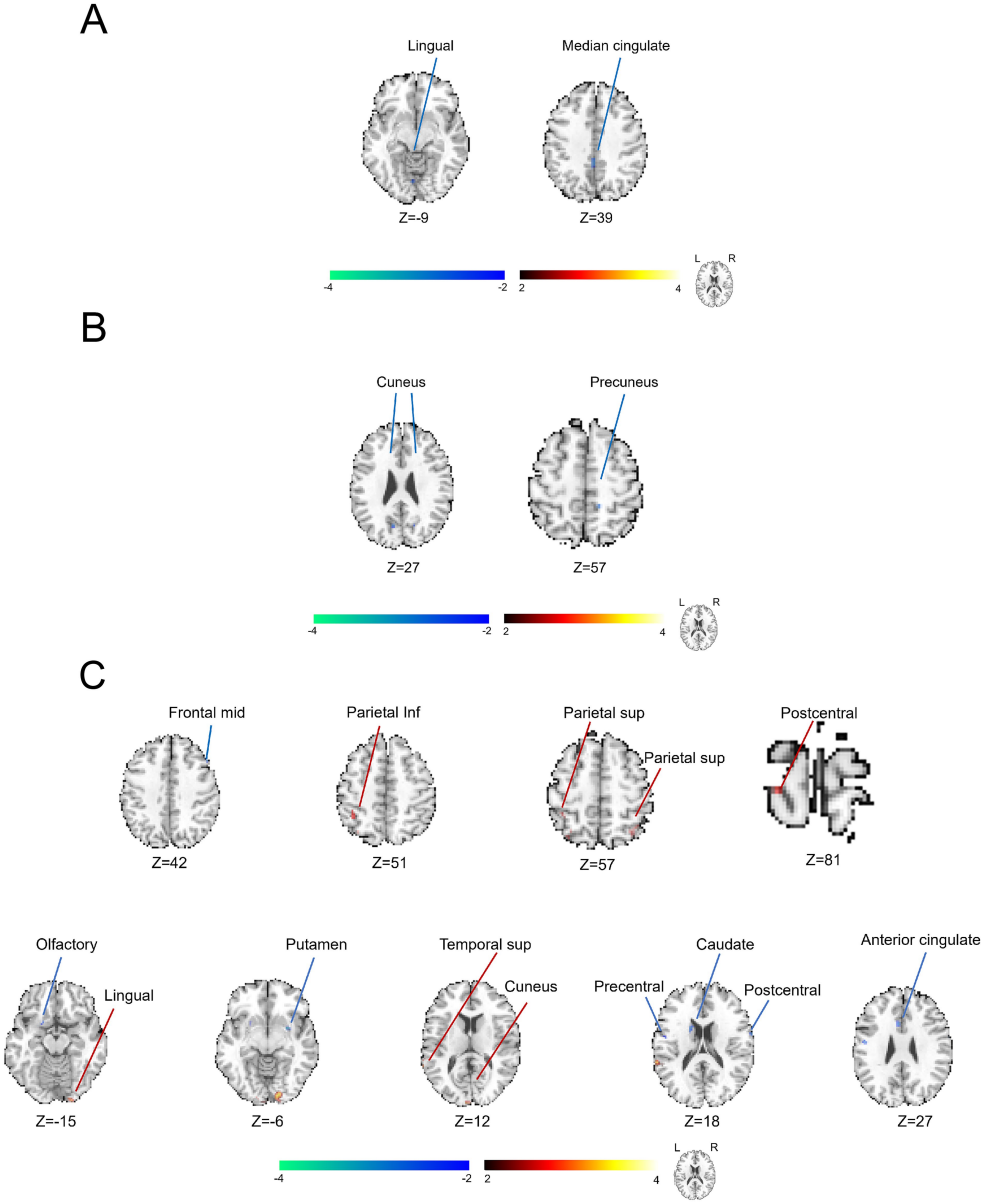
Utilization of KCC-ReHo analysis to characterize the brain functional alterations grouped by distinct severity of symptoms in the patients with CP/CPPS. **(A)** Comparison between the groups of severe and moderate conditions with symptoms. **(B)** Comparison between the groups of moderate and mild conditions with symptoms. **(C)** Comparison between the groups of severe and mild conditions with symptoms.

As demonstrated in Figure 3, we further displayed the brain functional abnormalities detected by the analysis of KCC-ReHo in the comparisons of different self-reported severity of symptoms. In Figure 3A and Supplementary Table S4, we found that brain regions of the right superior temporal gyrus and supramarginal gyrus revealed abnormally positive activation. In contrast, the brain regions of the right cuneus, right precuneus, left superior medial frontal region and left inferior parietal region (cluster > 5 voxels, GRF, *p* < 0.05) showed unusually negative activation between groups presenting with severe and mild prostate-like symptoms. In Figure 3B and Supplementary Table S5, we observed increased abnormal alterations in the right supramarginal gyrus and right middle temporal gyrus (cluster > 5 voxels, GRF, *p* < 0.05) between groups of moderate and mild prostate-like symptoms. Conversely, decreased abnormal alterations were noted in the left superior medial frontal region, left posterior cingulate cortex, right superior frontal region (area 2), left cuneus, left angular gyrus, right cuneus, left postcentral gyrus, left precuneus, right parahippocampal gyrus, and right lingual gyrus (cluster > 5 voxels, GRF, *p* < 0.05), suggesting reduced neural activity in the same comparison. In Figure 3C and Supplementary Table S6, in the comparison of severe and mild conditions with symptoms, we detected an increase in ReHo values within the right precuneus and a decrease in ReHo values were noted across several brain regions, including the right postcentral gyrus, left inferior frontal opercular cortex, right supramarginal gyrus, left middle frontal region (area 2), right inferior temporal gyrus, right superior frontal region (area 2), and the left supplementary motor area (cluster > 5 voxels, GRF, *p* < 0.05).

## Discussion

The CP/CPPS stands as a predominant health threat to men, posing a particularly significant health threat to the well-being of young men (Lee and Cho, 2017). In CP/CPPS, chronic pain is the most significant complaint and is most commonly reported in the perineum and suprapubic area, followed by the testicles, inguinal region, penis, and buttocks (Sevim et al., 2023). The elusive pathophysiology of this condition has constrained advancements in therapeutic development and prognostic accuracy. Previously, our research group has identified several abnormal brain functional alterations that potentially contribute to both the pathogenesis and the associated symptoms of CP/CPPS, as revealed through analysis of resting-state functional magnetic resonance imaging (fMRI) data (Ge et al., 2022; Ge et al., 2021). Analyzing each item in the NIH-CPSI, we identified significant yet variable differences among the recruited cohort of CP/CPPS patients, describing the heterogeneity within the group. As the symptom severities of CP/CPPS escalated, the observed functional brain abnormalities altered, indicating a potential correlation between the progression of the disease and the neurological changes. Hence, we initially launched a comprehensive discussion to establish a detailed protocol for categorizing functional brain alterations with different severity of symptoms.

The neural substrates that subserve the processing of pain perception, collectively called the pain matrix, encompass a network of key brain regions including the primary and secondary somatosensory cortices (SI and SII), ACC, lateral prefrontal cortex (lPFC), medial prefrontal cortex (mPFC), insula, supplementary motor area (SMA), and thalamus, which together facilitate the complex experience of pain (Tsuji et al., 2021; Zhang et al., 2022). As depicted in Figure 1, when compared to the healthy control group, we identified significant functional brain alterations in patients with CP/CPPS, which were predominantly localized to the brain regions of the pain matrix. The insula plays a pivotal role in the perception of injury, as well as in the cognitive and emotional dimensions of pain regulation (Yang et al., 2023). The amygdala, a central component of the limbic system, has been consistently implicated in the modulation of anxiety, depression, and fear learning (Ding et al., 2023). Under chronic pain conditions, the thalamus has been implicated as a critical hub in the neural circuitry underlying both spontaneous and evoked pain manifestations (Liu et al., 2021). The amygdala receives nociceptive signals from the brainstem and subsequently relays this information to the thalamus (Hsiao and Lin, 2022). The neural circuit between the thalamic reticular nucleus and lateral habenula was proven to modulate depressive-like behaviors in chronic stress and chronic pain conditions (Wang et al., 2023). In the mouse model of spared nerve injury (SNI), it was proved that the neural circuits connecting the insula to the amygdala and the insula to the thalamus potentially served as significant pathophysiological substrates for the development of hyperalgesia and depression-like behaviors in neuropathic pain (Chen et al., 2024). Therefore, our study demonstrated that the neurobiological alterations of these cortical and subcortical brain areas observed in CP/CPPS closely resemble those typically associated with chronic pain states (Ge et al., 2021).

In this research, we first illustrated the functional brain alterations with the separate symptom severity of CP/CPPS and discovered the diagnostic potential of DC value within the brain region of the right ACC. The ACC serves as a key cortical area for representing pain, predominantly engaged in encoding emotional pain information, which is integral to the affective dimension of the pain experience (Qiu et al., 2024). Furthermore, the ACC is critical in pain processing, as it integrates dual inputs from the medial pain pathway and the limbic system and sends projection fibers to the frontal lobe, thereby exerting its influence on the cognitive and emotional aspects of pain perception (Rolls, 2019). ACC is vital in pain perception, exhibiting consistent activities responding to noxious stimuli and hyperactivity in chronic pain conditions (Franciosa et al., 2024). In the mouse model of SNI, it was observed that the activation of excitatory neurons in the ACC lowered the mechanical pain threshold and shortened the response latency to heat-related pain in normal mice, whereas the inhibition of excitatory neurons in the ACC elevated the pain threshold in the murine model, which exhibited ACC might modulate the neuropathic pain in mice (Zhuang et al., 2021). Electroencephalography (EEG) unveiled that there was enhanced connectivity between the left dorsolateral prefrontal cortex and the right ACC within the beta-3 frequency band in women diagnosed with fibromyalgia (FM) (Alves et al., 2023). An increase in neuronal activity within the ACC may serve complementary roles in modulating the chronic pain state, for chronic pain has been shown to elevate both the baseline and noxious stimulus-induced neuronal firing rates in the ACC (Li et al., 2019). Thus, the fluctuations in DC values within the right ACC may serve as a promising biomarker for the diagnosis of CP/CPPS as well as for evaluating the severity of the condition.

By conducting a correlation analysis of NIH-CPSI items, our research team identified significant internal relationships that linked the severity of symptoms to the manifestation of pain symptoms. Pain is the core symptom that prompts patients to seek help (Romano et al., 2016). The pain of CP/CPPS exerted a more significant influence on QoL compared to urinary symptoms in patients with pelvic disorders, and the severity and frequency of pain are more critical factors than its localization or type (Wagenlehner et al., 2013). Pain catastrophizing was strongly linked to self-reported pain (Romano et al., 2016). Pain catastrophizing was exposed to influence pain perception by altering attention and anticipation of pain (Terry et al., 2022). In chronic pain conditions, a connection was established between pain catastrophizing and brain regions closely associated with pain perception like the S1, S2, anterior insula, ACC, and thalamus, and/or modulation like the dorsal lPFC (Galambos et al., 2019). In the patients of CP/CPPS, it was reported that a high prevalence of pain catastrophizing was observed (Huang et al., 2020). In the patients of FM, pain catastrophizing was observed to be associated with amplified baseline activity in the S1 – insula by the detection of fMRI (Aman et al., 2018). Likewise, the outcomes of our research provided neuroscientific insight into the association between alterations in the central nervous system and self-reported clinical scale of symptoms.

## Data Availability

The datasets used and analyzed in the current study are available from the corresponding author upon reasonable request.

## Footnotes

^1^ http://restfmri.net/forum/restplus

^2^ http://www.fil.ion.ucl.ac.uk/spm/
