# Supplementary material for: The characteristics of brain function alterations in patients with chronic prostatitis/chronic pelvic pain syndrome across varying symptom severities evaluated by NIH-CPSI
Source: Front Neurosci. 2025 Feb 26;19:1511654. doi: 10.3389/fnins.2025.1511654 (PMC11897570; doi:10.3389/fnins.2025.1511654)
Supplement: Supplementary file 1 [file Table_1.docx]

**Supplement data**

**sTable 1. The brain regions of abtabnormal activities between the severe and moderate conditions with symptoms assessed by the analysis of DC**

| Contrast Name | |  |  | MNI Coordinates | | |
| --- | --- | --- | --- | --- | --- | --- |
|  | Region Label | Extent | t-value | x | y | z |
| Positive | Temporal_Sup_L | 50 | 3.891 | -63 | -39 | 21 |
|  | Lingual_R | 68 | 3.846 | 18 | -99 | -12 |
|  | Cuneus_L | 69 | 3.267 | -6 | -99 | 12 |
|  | Parietal_Sup_R | 51 | 3.100 | 39 | -57 | 57 |
|  | Postcentral_L | 12 | 3.020 | -21 | -30 | 81 |
|  | Parietal_Inf_L | 28 | 2.870 | -39 | -48 | 51 |
|  | Parietal_Sup_L | 14 | 2.779 | -33 | -69 | 54 |
| Negative | Olfactory_L | 23 | -3.839 | -21 | 9 | -15 |
|  | Precentral_L | 37 | -3.724 | -48 | -9 | 30 |
|  | Putamen_R | 26 | -3.535 | 30 | 0 | -6 |
|  | Frontal_Mid_2_R | 23 | -3.176 | 48 | 27 | 42 |
|  | Cingulate_Ant_L | 19 | -3.001 | -6 | 15 | 27 |
|  | Caudate_L | 17 | -2.905 | -18 | 3 | 18 |
|  | Postcentral_R | 23 | -2.904 | 66 | 0 | 21 |

**sTable 2. The brain regions of abnormal activities between the moderate and mild conditions with symptoms assessed by the analysis of DC**

| Contrast Name | |  |  | MNI Coordinates | | |
| --- | --- | --- | --- | --- | --- | --- |
|  | Region Label | Extent | t-value | x | y | z |
| Negative | Cingulate_Mid_L | 28 | -2.929 | 0 | -42 | 39 |
|  | Lingual_L | 11 | -2.863 | -3 | -75 | -9 |

**sTable 3. The brain regions of abnormal activities between the** **severe and mild conditions with symptoms assessed by the analysis of DC**

| Contrast Name | |  |  | MNI Coordinates | | |
| --- | --- | --- | --- | --- | --- | --- |
|  | Region Label | Extent | t-value | x | y | z |
| Negative | Cuneus_L | 15 | -3.030 | -9 | -69 | 24 |
|  | Precuneus_R | 12 | -2.893 | 12 | -42 | 57 |
|  | Cuneus_R | 10 | -2.703 | 18 | -69 | 27 |

**sTable 4. The brain regions of abnormal activities between the severe and moderate conditions with symptoms assessed by the analysis of ReHo**

| Contrast Name | |  |  | MNI Coordinates | | |
| --- | --- | --- | --- | --- | --- | --- |
|  | Region Label | Extent | t-value | x | y | z |
| Positive | Precuneus_R | 37 | 3 | 6.000 | -60 | 54 |
| Negative | Postcentral_R | 47 | -5.157 | 60 | 0 | 30 |
|  | Frontal_Inf_Oper_L | 56 | -4.390 | -48 | 9 | 12 |
|  | SupraMarginal_R | 17 | -3.266 | 60 | -27 | 36 |
|  | Frontal_Mid_2_L | 30 | -3.231 | -33 | 54 | 30 |
|  | Temporal_Inf_R | 24 | -3.132 | 48 | -24 | -21 |
|  | Frontal_Sup_2_R | 10 | -3.103 | 24 | 63 | 21 |
|  | Supp_Motor_Area_L | 11 | -2.721 | -6 | -12 | 57 |

**sTable 5. The brain regions of abnormal activities between the moderate and mild conditions with symptoms assessed by the analysis of ReHo**

| Contrast Name | |  |  | MNI Coordinates | | |
| --- | --- | --- | --- | --- | --- | --- |
|  | Region Label | Extent | t-value | x | y | z |
| Positive | Temporal_Sup_R | 22 | 2.749 | 60 | -48 | 21 |
|  | SupraMarginal_R | 14 | 2.703 | 60 | -24 | 36 |
| Negative | Cuneus_R | 51 | -3.249 | 15 | -69 | 21 |
|  | Precuneus_R | 51 | -2.640 | 15 | -57 | 39 |
|  | Frontal_Sup_Medial_L | 36 | -3.171 | -9 | 66 | 24 |
|  | Parietal_Inf_L | 40 | -3.151 | -42 | -30 | 39 |

**sTable 6. The brain regions of abnormal activities between the** **severe and mild conditions with symptoms assessed by the analysis of DC**

| Contrast Name | |  |  | MNI Coordinates | | |
| --- | --- | --- | --- | --- | --- | --- |
|  | Region Label | Extent | t-value | x | y | z |
| Positive | SupraMarginal_R | 12 | 3.261 | 60 | -48 | 27 |
|  | Temporal_Mid_R | 58 | 3.228 | 60 | -48 | 9 |
| Negative | Frontal_Sup_Medial_L | 69 | -4.583 | -9 | 66 | 21 |
|  | Cingulate_Post_L | 29 | -4.437 | -15 | -45 | 6 |
|  | Frontal_Sup_2_R | 49 | -4.016 | 24 | 63 | 21 |
|  | Cuneus_L | 88 | -3.843 | -9 | -63 | 24 |
|  | Angular_L | 16 | -3.794 | -30 | -51 | 36 |
|  | Cuneus_R | 27 | -3.377 | 18 | -69 | 21 |
|  | Postcentral_L | 15 | -3.184 | -54 | -21 | 27 |
|  | Precuneus_L | 12 | -3.034 | -9 | -45 | 45 |
|  | ParaHippocampal_R | 27 | -3.012 | 18 | -42 | -9 |
|  | Lingual_R | 26 | -2.708 | 6 | -69 | -3 |
